# RNA-Seq Mapping and Detection of Gene Fusions with a Suffix Array Algorithm

**DOI:** 10.1371/journal.pcbi.1002464

**Published:** 2012-04-05

**Authors:** Onur Sakarya, Heinz Breu, Milan Radovich, Yongzhi Chen, Yulei N. Wang, Catalin Barbacioru, Sowmi Utiramerur, Penn P. Whitley, Joel P. Brockman, Paolo Vatta, Zheng Zhang, Liviu Popescu, Matthew W. Muller, Vidya Kudlingar, Nriti Garg, Chieh-Yuan Li, Benjamin S. Kong, John P. Bodeau, Robert C. Nutter, Jian Gu, Kelli S. Bramlett, Jeffrey K. Ichikawa, Fiona C. Hyland, Asim S. Siddiqui

**Affiliations:** 1 Life Technologies, Foster City, California, United States of America; 2 Indiana University School of Medicine, Indianapolis, Indiana, United States of America; 3 Life Technologies (Ambion), Austin, Texas, United States of America; Washington University in Saint Louis, United States of America

## Abstract

High-throughput RNA sequencing enables quantification of transcripts (both known and novel), exon/exon junctions and fusions of exons from different genes. Discovery of gene fusions–particularly those expressed with low abundance– is a challenge with short- and medium-length sequencing reads. To address this challenge, we implemented an RNA-Seq mapping pipeline within the LifeScope software. We introduced new features including filter and junction mapping, annotation-aided pairing rescue and accurate mapping quality values. We combined this pipeline with a Suffix Array Spliced Read (SASR) aligner to detect chimeric transcripts. Performing paired-end RNA-Seq of the breast cancer cell line MCF-7 using the SOLiD system, we called 40 gene fusions among over 120,000 splicing junctions. We validated 36 of these 40 fusions with TaqMan assays, of which 25 were expressed in MCF-7 but not the Human Brain Reference. An intra-chromosomal gene fusion involving the estrogen receptor alpha gene ESR1, and another involving the RPS6KB1 (Ribosomal protein S6 kinase beta-1) were recurrently expressed in a number of breast tumor cell lines and a clinical tumor sample.

## Author Summary

Advances in sequencing technology are enabling detailed characterization of RNA transcripts from biological samples. The fundamental challenge of accurately mapping the reads on transcripts and gleaning biological meaning from the data remains. One class of transcripts, gene fusions, is particularly important in cancer. Some gene fusions are prominent markers in leukemia, prostate, and other cancers and putatively causative in certain tumor types. We present a set of new RNA-Seq analysis techniques to map reads, and count expression of genes, exons and splicing junctions, especially those that give evidence of gene fusions. These tools are available in a software package with a straightforward graphical user interface. Using this software, we called and validated several gene fusions in a breast cancer cell line. By testing the presence of these fusions in a larger population of tumor cell lines and clinical samples, we found that two of them were expressed recurrently.

## Introduction

The transcriptome comprises the set of all transcripts in a cell and their quantity at a specific stage and time. RNA-Seq enables hypothesis-neutral investigation of the expression of the transcripts including non-coding RNA and viruses [1]. RNA-Seq provides advantages over microarray technology such as the detection of novel transcripts (both truly novel as well as those arising from alternative splicing) and sensitivity over a greater range of expression [2]. Methods to more comprehensively analyze RNA sequencing data are being developed, with particular focus on normalization of differential gene expression, annotation of the transcriptome, and characterization of the splicing junctions [3]–[12]. Paired-end RNA-Seq further enhances quantification of alternative transcripts [13]–[16]. Analysis of tissue and single-cell-specific RNA is revealing cellular gene expression diversity and phenotypy [17]–[19].

Gene fusions arise from mutations including translocations, deletions, inversions, or trans-splicing. Fusion genes are thought to cause tumorigenesis by over-activating proto-oncogenes, deactivating tumor suppressors, or altering the regulation and/or splicing of other genes which lead to defects in key signaling pathways [20]. Fused RNAs are found to occur in significantly higher frequency in cancer than in matched benign samples and may be potential biomarkers [21]. For example, 95% of patients with clinical chronic myeloid leukemia (CML) express the BCR-ABL gene fusion in their leukemia cells due to a reciprocal translocation between the long arms of chromosomes 9 and 22 [22], [23]. BCR-ABL is also found to be a factor in 30% to 50% of adult acute lymphoblastic leukemia cases [24]. Imatinib is a specific tyrosine kinase inhibitor targeting BCR-ABL and is an effective treatment for CML [25], [26]. Gene fusions are also detected repeatedly in other tumors. Examples include ETV6-NTRK3 in mesoblastic nephroma, congenital fibrosarcoma, and breast carcinoma [27]–[29]. MYB-NFIB in head and neck tumors [30], TMPRSS2-ERG/ETS in prostate cancer [31]–[34], and EML4-ALK in lung cancer [33], [35]. Most lung tumors with ALK rearrangements are shown to shrink and stabilize when patients are given the ALK inhibitor Crizotinib [36].

Hypothesis-neutral gene fusion detection with RNA-Seq was recently demonstrated by different groups [37]–[46]. For example, the FusionSeq software uses paired-end reads to find candidate fusions, and applies a set of filtration modules to remove false positive candidates [41]. FusionSeq applies misalignment filters for large- and small-scale homology, low complexity repetitive regions, and mitochondrial genes particularly considering reads that fall on SNP regions or on RNA edited transcripts that may cause misalignments. deFuse guides a dynamic programming based spliced read detection module with paired-end alignments [42]. Both of these methods reply upon paired-end alignments as the initial evidence and apply spliced read mapping on the candidate regions. PERAlign relies upon mapping spliced reads to the whole genome first and then guiding them with paired-end alignments [38].

In this study, we describe a new method which considers spliced-read and paired-end alignments independently from each other, enabling detection of fusions from single fragment or paired-end experiments. We also introduce techniques for mapping of spliced-reads to a suffix array based virtual gene fusion reference with annotation-aided pairing rescue and methods for quality assessment of alignments and splice junctions. We tested our analysis tool by calling the exon/exon junctions and gene fusions from data generated by sequencing three paired-end RNA-Seq libraries, each with two technical replicates. We also compared our results to TopHat and FusionSeq software on the MCF-7 sample. Next we validated candidate MCF-7 gene fusions using TaqMan® assays and showed that 90% of the calls were valid and over 65% were specific to MCF-7. We also identified what appears to be an early breakpoint bias at the 5′ fused genes. Finally, we surveyed a subset of MCF-7 and UHR fusions on a panel of breast cancer cell lines and discovered evidence for recurrence.

## Results

### A combined strategy to detect splice junctions and fusion breakpoints

We prepared strand-specific, paired-end RNA libraries from the Universal Human Reference (UHR), the Human Brain Reference (HBR), and the breast cancer cell line MCF-7 using the Total RNA-Seq kit from Applied Biosystems. These RNA libraries were sequenced using ligation-based high throughput SOLiD™ system [47]. Fragments were gel-selected for insert sizes between 100–200 base pairs (Figure S1 in Text S1). Using a new transcriptome alignment pipeline in which each pair of reads is mapped to genome, junction, exon and filter references and paired with a pairing quality value (PQV), we obtained total of 580 million read pairs that were confidently mapped to the human genome (Table S1 in Text S2). Histograms of gene expression showed a wide range of distribution, and average R^2^ correlation of gene expression between replicates ranged from 0.95 to 0.96 (Figures S2, S3 in Text S1).

**Figure 1:**
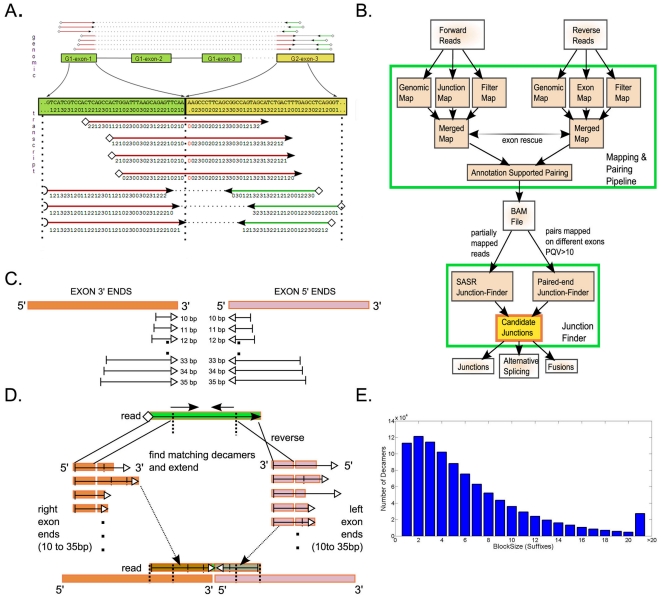
RNA-Seq mapping and splice junction detection methodology. **A.** Four reads that span (spliced single reads), and three reads that bridge (paired-end reads) the junction are shown. The top chart shows a bird's eye view of the genomic alignments detected for seven pairs of reads between the two exons. Areas of the read highlighted in red correspond to colors that do not align to a genomic reference, and dots in the reference are unknown colors/bases. **B.** Mapping pipeline is reviewed in the Methods sections. Candidate junctions correspond to a sparse graph of junction evidences. After the candidates are found, splice junction and fusion predictions are made with optional quality thresholds. **C.** As a first step in SASR, 10 to 35 bp ends from each end of the exon are stored in two lexicographical dictionaries. Stored suffix starts are shown as a vertical stop and end with empty triangles. **D.** 10 base pairs from the left and right ends of the read (decamers) are searched in the 3′ and 5′ end dictionaries, respectively, with a binary string search. Decamers are matched without mismatches. Matching decamers are extended as possible (with up to two mismatches) to determine whether they cover the entire suffix. Mismatches are illustrated as vertical lines. Up to ten bases are clipped from the ends of the reads until a matching read is found. **E.** Decamer block size frequency in the hg18 RefSeq database.

Splice junctions were discovered by combining three approaches: (1) BRIDGE evidence found by paired-end reads in which the forward read maps on an exon and the reverse read maps on another exon with a PQV above a confidence threshold; (2) SPAN evidence found by single reads (of paired-end reads) in which the read alignment spans the breakpoint of a set of known and putative splice junctions; (3) Fusion SPAN evidence found by fusion alignments spanning hypothetical breakpoints of any two exons discovered using the SASR aligner which assesses all exon-exon combinations in the genome (Figure 1). Using this strategy, for each sample, we identified an average of 133,000 RefSeq and 15,315 non-RefSeq (putative) splice junctions and 5 to 56 candidate fusion breakpoints (Table 1 and Table S1 in Text S2).

**Table 1:** Mapping and splicing statistics for paired-end runs.

Dataset	#Confidently Aligned Pairs	#Known Splice	#Putative Splice	#Putative Fusion
UHR-1	79,654,007	127,987	9,025	5
UHR-2	113,699,316	136,839	14,365	13
HBR-1	89,066,940	129,031	8,709	8
HBR-2	130,521,674	138,718	14,204	14
MCF-7-1	79,654,007	123,442	17,373	40
MCF-7-2	86,796,592	120,503	19,437	56

**Notes:** Confidently aligned pairs was defined as primary alignments with PQV>10. 120 and 150 refer to insert size of RNA library. MCF-7 and MCF-7 -2 libraries were prepared separately from the same lot. Known splicing events are found in RefSeq database whereas putative splicing events were not.

To assess the performance of mapping quality values generated with the system, we compared fold-change ratio (Log2 [UHR/HBR]) of gene RPKM values with gene expression assays from the MicroArray Quality Control (MAQC) project [48], [49]. We compared correlation of four different PQV (1, 10, 20 and 40) thresholds with the assays (Figure S4 in Text S1). Pearson correlations of data from TaqMan assays with that of the data from the SOLiD system were not significantly different between PQV thresholds. The slope (*m*) of the regression fits, however, was significantly affected by the threshold settings. As PQV is increased from 1 to 40, the slope increased dramatically from 0.77 to 0.88, indicating significantly greater accuracy compared to a “gold standard” qPCR method. The increase in accuracy is likely a result of increased specificity. Essentially, the log ratio dynamic range increases with increasing PQV settings (Figure S5 in Text S1). RPKM distributions show an increase in low-end signal for lower PQV. If this increase in “sensitivity” represents additional noise, it can contribute to a loss of accuracy in the fold change calculations. The increase in the low end suggests that these reads may be spurious (Figure S6 in Text S1).

### Parameter stringency and quality assessment

**Figure 2:**
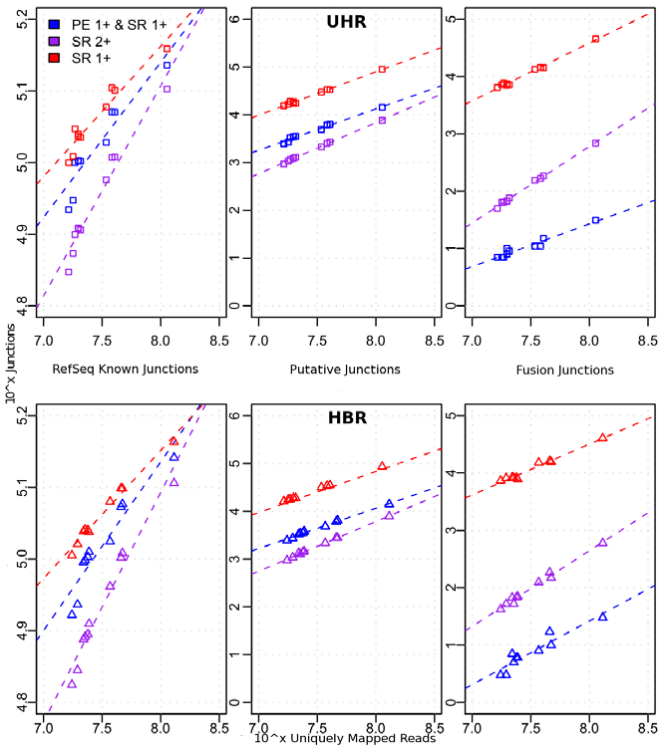
Combined evidence improves specificity of splice and fusion detection. Scatterplots show the increasing mapped coverage (x-axis) versus Left: Known RefSeq junctions; Middle: Putative junctions; Right: Fusion junctions. Top track shows results for UHR and bottom track for HBR. Three different evidence thresholds were compared: 1) red line: one SPAN (SR) evidence required for junction call, 2) magenta line: two SPAN (2-SR) evidences required for junction call, and 3) blue line: one SPAN and one BRIDGE evidence (1-SR-1-PE) required for junction call.

In order to find optimal filters for detecting splice junctions with our combined approach, we compared three quality thresholds using data from the UHR and HBR barcoded libraries: (1) one SPAN evidence (1-SR), (2) two unique SPAN evidences (2-SR), and (3) one BRIDGE and one SPAN evidences (1-PE-1-SR). In addition to these tested thresholds, we applied default filter of choosing only primary alignments with PQV>10. The results, as illustrated in Figure 2, suggest an increased number of false positives for 1-SR evidence even though it may have greater sensitivity. 1-PE-1-SR threshold reduced false positives especially for fusions, generating less calls than 2-SR threshold. For the analyses described later in the text, 1-PE-1-SR threshold was chosen for calling splice junctions and 2-PE-2-SR for calling gene fusions. On average, 82% of junctions identified in the libraries were present in the RefSeq database. 84% of these known junctions and 26% of the putative junctions were shared between at least two of the three libraries (Figure S7 in Text S1). The highest number of library-specific known junctions was observed in HBR, and the highest number of library-specific putative junctions was observed in MCF-7.

**Figure 3:**
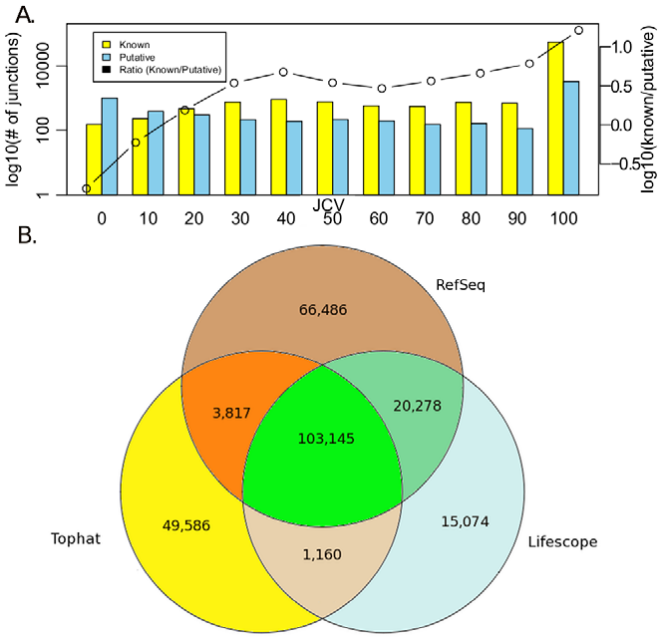
Improvements by junction confidence value and comparison to TopHat. **A.** Logarithms of number of known and putative junctions are shown with yellow and blue bars respectively. The ratio of known over putative is shown with dashed line. Dataset consisted of 64,000 sample UHR junctions called with default thresholds. **B.** TopHat and Lifescope candidate calls were compared to each other and also to RefSeq database. TopHat junctions were filtered with score>5, and Lifescope junctions were filtered with 1-SR-1-PE threshold (requiring one span and one bridge evidence).

Next, we formulated a Junction Confidence Value (JCV) and investigated its utility to identify true versus false junctions. Details of JCV and its formulation are explained in supplementary methods in Text S1. One type of false positive fusion junction is likely called between highly expressed exons for which the random chance of encountering a misalignment or mispairing is elevated. Homology between highly expressed genes would also increase this type of false positive. We created JCV to test the quantity and quality of BRIDGE evidences when compared to an ‘error expectation metric’. This metric is defined as the estimation of the null hypothesis of encountering a random junction between the two exons. Increasing JCV increased known/putative junction ratio which was predictive of the false discovery rate and at the same time distinguished significant number of novel junctions to either lower or higher score bins (Figure 3A). Known/putative ratios ranged from 0.15 for JCV cutoff of 0; 3 for JCV cutoff of 50 and 16 for JCV cutoff of 100. In order to test the sensitivity of JCV, we simulated 1,000,000 junctions based on a combination parameter model of true junction expression ratio and false junction misalignment ratio. True positive rate (TPR) and false positive rate (FPR) were calculated by comparing whether a called junction was real (Figures S8, S9, S10 in Text S1). These simulations showed that JCV was predictive of true junction calls and higher JCV thresholds resulted in much less FPR and slightly less TPR. We performed a separate simulation of gene fusion detection using reads from a DH10B (E.coli) DNA sequencing experiment where introns, exons and a gene model were simulated to make the data similar to RNA-Seq experiments. Our algorithm was able to detect 86 out of 93 simulated fusions in this experiment (Figure S11 in Text S1).

Next, we ran TopHat (v1.3.0) on the MCF-7-1 dataset by using default paired-end parameters for color space. TopHat reported 124,236,156 mapped reads of which 33,634,800 were properly paired whereas LifeScope reported 404,901,929 mapped reads, of which 300,341,259 pairs were mapped to the same chromosome and 158,050,096 were properly paired (Table S1 in Text S2). Of note, TopHat allows 2 mismatches on mapped reads by default and does not report pairs of reads mapped across different chromosomes. Next we identified appropriate score threshold for calling TopHat junctions. For each junction found, TopHat reports a score which corresponds to the number of reads that span the junction. TopHat reported 1,391,319 total junctions without any score filter and with score>5 threshold this number reduced to 53,402 (Figure S12 in Text S1). We used TopHat candidate junctions with score>5 for comparison to RefSeq known and Lifescope candidate junctions (Figure 3B). There is some evidence that score>10 may yield more specific results for TopHat (Figure S12 in Text S1). Of note, known (RefSeq) junctions called by TopHat dropped from 129,316 (score>0) to 106,962 (score>5). These results suggest that TopHat detects a large number of putative novel junctions yet is not as sensitive when distinguishing false positives. LifeScope detected 15,074 putative novel junctions between known exons of the same gene that weren't called by TopHat. We could distinguish that more than half of these ‘LifeScope-only’ junctions were likely true positives by looking at their JCV; 3,520 had JCV = 0 and 8,481 had JCV = 100 with the rest having scores between 0 and 100.

### Detection and validation of fusion transcripts in the MCF-7 cell line

Using the combined BRIDGE&SPAN approach described above on the UHR sample, we called and validated previously reported gene fusions including BCR-ABL1, GAS6-RASA3, ARFGEF2-SULF2, NUP214-XKR3 and BAT3-SLC44A4 [37], [39], [40]. These fusions were not described in the literature for HBR, and as expected were not identified in the HBR samples sequenced. In MCF-7, a total of 40 putative fusions were identified in the first sequencing run (50×25 paired-end), of which 26 were detected again in a second run (75×35 paired-end) out of a total 56 fusion calls (Table S2 in Text S2). We also analyzed first MCF-7 sequencing dataset using FusionSeq (Sboner et. al.). Six of the forty gene fusions identified by LifeScope were also called by this software. FusionSeq's confidence value (RESPER) for these calls ranged from 1.15 to 4.53. Of importance, the ribosomal filter and single read validation module of FusionSeq (version 0.7) did not handle color space data or data with different read length pairs adding to 5807 total calls with RESPER>1 (Table S3 in Text S2).

Based on the calls from the first MCF-7 sequencing experiment, we prepared 40 TaqMan fusion assays and run them on the UHR, HBR, and MCF-7 samples along with the prostate cell line PC-3 as an additional control. 36 (90%) of the fusions were validated with the assays and 25 (63%) were found to be specific to MCF-7 and UHR (Table 2 and Table S4 in Text S2). To note, 19 of these “specific” fusions were called with our algorithms in the second run of MCF-7. JCV values correlated with whether a fusion was called again, and also with the number of unique start points (Figure S13 in Text S1).

**Table 2:** Validated MCF-7 gene fusions and TaqMan expression ratios.

5′ Gene Exon	Chr	3′ Gene Exon	Chr	Distance	MCF-7	UHR	HBR	PC-3
ARFGEF2-1	20	SULF2-3	20	Inverted	20.6	24.2	40.0	39.7
SLC25A24-4	1	NBPF6-16	1	Inverted	23.9	27.9	40.0	40.0
USP31-1	16	CRYL1-4	13	Inter-chr	27.5	31.8	40.0	40.0
TBL1XR1-1	3	RGS17-2	6	Inter-chr	26.1	30.6	40.0	40.0
TAF4-1	20	BRIP1-5	17	Inter-chr	25.6	29.2	40.0	40.0
RPS6KB1-6	17	DIAPH3-30	13	Inter-chr	22.6	26.1	40.0	36.7
BCAS4-1	20	BCAS3-24	17	Inter-chr	21.3	25.3	40.0	40.0
AHCYL1-1	1	RAD51C-10	17	Inter-chr	31.0	34.8	40.0	40.0
ABCA5-4	17	PPP4R1L-4	20	Inter-chr	26.1	29.9	40.0	40.0
C16orf45-1	16	ABCC1-15	16	641567	25.3	29.2	40.0	40.0
C16orf62-8	16	IQCK-10	16	264613	26.7	30.5	40.0	40.0
CXorf15-1	X	SYAP1-2	X	−51362	29.1	32.7	40.0	40.0
MYO6-1	6	SENP6-15	6	−70841	28.4	31.9	40.0	40.0
RPS6KB1-2	17	TMEM49-11	17	−72316	24.4	28.4	40.0	39.8
SMARCA4-7	19	CARM1-2	19	−81642	29.9	33.1	40.0	40.0
POP1-2	8	MATN2-15	8	−86928	28.5	31.8	40.0	40.0
GATAD2B-1	1	NUP210L-28	1	−107321	28.3	32.4	40.0	40.0
ESR1-2	6	C6orf97-7	6	−116116	32.3	35.0	40.0	40.0
ESR1-2	6	C6orf97-6	6	−128831	25.2	29.1	40.0	40.0
DEPDC1B-7	5	ELOVL7-8	5	−118895	25.6	29.0	39.8	40.0
GCN1L1-2	12	MSI1-12	12	−157216	25.3	28.2	40.0	39.8
ATXN7L3-1	17	FAM171A2-4	17	−158568	24.8	28.3	40.0	40.0
SYTL2-1	11	PICALM-20	11	−217187	26.7	30.7	40.0	40.0
ADAMTS19-1	5	SLC27A6-10	5	−432137	26.5	31.3	40.0	40.0
ADAMTS19-2	5	SLC27A6-10	5	−433412	25.8	30.5	40.0	40.0

**Notes:** Each exon name (gene name-dash-exon-order) was obtained from RefSeq database. Inverted fusions are on same chromosome but different strands. Last four columns show the Cycle Threshold (CT) value in TaqMan assays. Lower CT values indicate higher expression.

Real-time PCR Cycle Threshold (CT) values showed that each of the MCF-7 gene fusions was expressed in UHR with around ten-fold less expression (Table 2). This suggests that MCF-7 or one of its parent or sister cell lines is very likely part of the UHR pool. According to the information provided by the supplier, UHR RNA is prepared from a pool of ten different cancer cell lines, one of which is an ‘adenocarinoma, mammary gland’. MCF-7 is an adenocarcinoma cell line from mammary gland. From the RNA-Seq calls, nine of the MCF-7 gene fusions were detectable in UHR with ∼200 million confidently mapped reads whereas these fusions were detectable in MCF-7 with only ∼80 million reads. It is likely that deeper sequencing of the UHR pool would have identified the remaining fusions.

**Figure 4:**
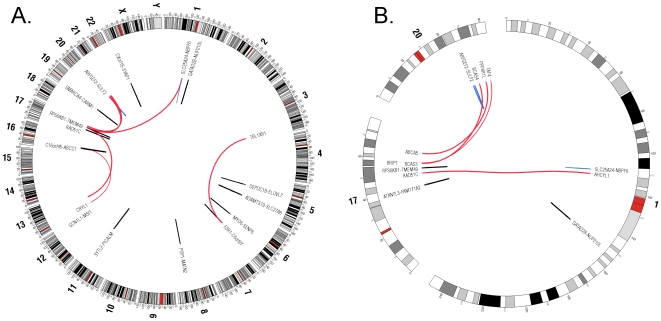
Localization of gene fusions on specific chromosomal regions. **A.** Whole genome and **B.** Chr 1, 17 and 20 gene fusions circular graph. Red lines represent inter-chromosomal gene fusions, blue lines represent inverted intra-chromosomal and black lines represent same-strand intra-chromosomal fusion events. Graphs were drawn with Circos software [61].

Many MCF-7 fusions were between genes in three bands of Chr 1, Chr 17 and Chr 20 (Figure 4). These bands were previously described as rearrangement “hot-spots” [50]. Of the total 11 inter-chromosomal or inverted intra-chromosomal fusions, five had premature stop codons (not in frame), while six were in frame. Two of the fusions were alternatively spliced including the fusion from the second exon of ESR1 to the sixth and seventh exon of C6orf97, and the fusion from the first and second exon of ADAMTS19 to the tenth exon of SLC27A6. We also found several new intra-chromosomal gene fusions mostly between adjacent or neighboring genes (Table S4 in Text S2).

**Figure 5:**
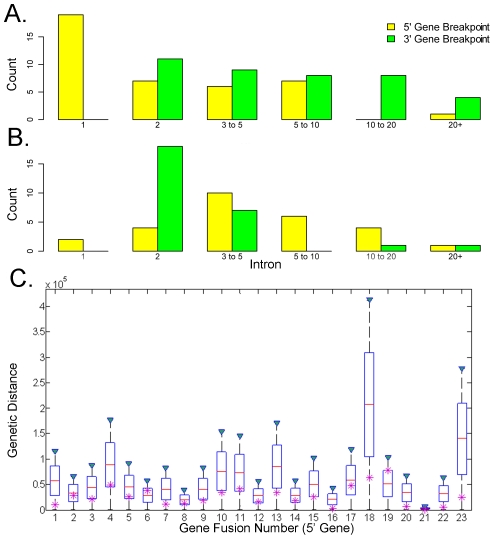
Fusion breakpoints are biased to 5′ end of the genes. Histogram of order of 5′ (yellow) and 3′ (green) intron breakpoints for **A.** MCF-7, **B.** UHR and HBR combined gene fusions. Breakpoint is inferred to happen at the intron (X axis) following the exon that is fused. Y axis shows the count of breakpoints that are inferred to happen at numbered intron. **C.** Boxplot of the distribution of simulated gene fusion locations for each of the 23 genes in which a fusion was observed. Magenta star marks the location of the observed fusion, relative to the 5′ exon. 23 fusions correspond to the gene fusions from Table 2 (except for *ESR1- C6orf97*, and *ADAMTS19- SLC27A6* alternatively spliced fusions merged into single data points).

We observed an enrichment of fusions for which the breakpoints were in the first intron of a gene, a similar bias explained also in Inaki et al., 2011. This pattern was not observed for the UHR and HBR samples (Figure 5A–B). On average, first introns in the RefSeq database (hg18) constitute 22% of a gene. We asked whether the large intron size alone might explain the breakpoint bias at the 5′ introns. We used a parametric bootstrap approach to test the hypothesis that gene fusions are more likely to occur towards the 5′ end of a gene; for example, after the first exon. Assuming that the breakpoint was in the middle of the intron following the fused exon, we considered breakpoints for 23 fusions from Table 2 (omitting multiple splices for ESR1 and ADAMTS19). We simulated 100,000 gene fusion locations in these 23 genes from a uniform distribution within the gene. We normalized the location of the real gene fusions by gene length (defined as the distance between the start of the first exon and the end of the last exon). We calculated the mean fusion location of the 23 genes, in the observed fusions and in the simulated fusions. In the real fusions, the mean insert location was 0.2587 (26% of the length of the gene, Figure 5C). In 100,000 simulated sets of 23 fusions, the mean was 0.5 and the standard deviation was 0.06. Only three in 100,000 of the simulated sets of fusions had a value less than 0.2587. Thus the observed location of the gene fusions is statistically significantly biased towards the 5′ end of the gene, with a p-value estimated at 3×10^−5^.

### Survey of UHR and MCF-7 fusions in cell lines and clinical tumor samples

**Figure 6:**
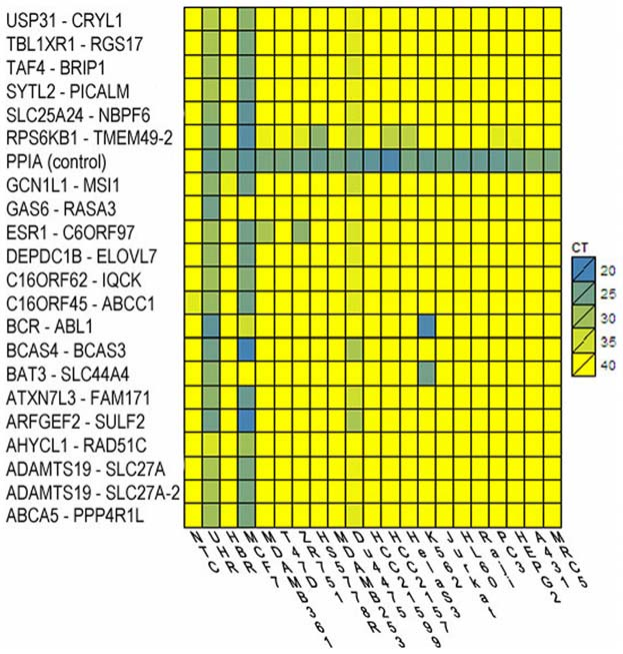
Screening of fusion assays in cancer cell lines reveal recurring fusions. Heat map of the expression of selected gene fusions (rows) in 20 samples including 18 cancer cell lines (columns). Lower cycle threshold (CT) indicates a higher level of expression and is highlighted in blue. High CT (max 40, yellow) indicates no expression. PPIA is used as positive control and non template control sample (NTC) as negative control.

To test recurrence, we selected 24 fusions from UHR and MCF-7 and investigated their expression in 20 cancer cell line samples (Figure 6). UHR fusions BCR-ABL1 and BAT3-SLC44A4 were found expressed in the myelogenous leukemia cell line K562 but with eightfold higher expression than in UHR. GAS6-RASA3 fusion was expressed only in UHR. Most of the fusions in MCF-7 were also expressed at a low level in the Du4475 cell line with a higher CT value (>35 for most cases). Both MCF-7 and Du4475 cell lines are traced to a 69/70-year old Caucasian female from Georgetown, but contamination, mixing, mislabeling, or differences in culturing may have caused the observed expression.

Two of the intra-chromosomal gene fusions were expressed in multiple samples: ESR1-C6ORF97 and RPS6KB1-TMEM49. The first of these fusions, between the estrogen receptor alpha gene ESR1 and its neighboring gene C6ORF97 on Chr 6 was expressed in two other ER+ breast cancer cell lines in addition to UHR, MCF-7, and Du4475. This fusion may have occurred due to an inversion or rearrangement, as normally the ESR1 gene is downstream of C6ORF97 on the genome (on the same strand, 128,831 base pairs apart); yet the fusion junction was observed to be from the second exon of ESR1 to the sixth exon of C6ORF97, in the reverse order of expected transcription. We noted that these two genes were considerably expressed in MCF-7 (RPKM 16 and 46 average), though not expressed at all in HBR (RPKM<0.5), and weakly expressed in UHR (RPKM 1 and 1.8). The second recurring fusion, RPS6KB1-TMEM49, was found expressed in four cancer cell lines including HCC2157 and HelaS3. We further tested the presence of 24 candidate fusions in cDNA from 48 Clinical Samples of Normal and Breast Tumors (Origene). We found ESR1-C6ORF97 expressed in one ER+ tumor, and none of the other fusions were expressed.

## Discussion

RNA-Seq allows interrogation of known and novel transcript expression and discovery of gene fusions. We describe a new suffix array algorithm to find fusion breakpoint spanning reads in a hypothesis-neutral fashion. We combine this algorithm with a new paired-end mapping approach to detect gene fusions sensitively and reliably. Our mapping method works with a predefined set of exon boundaries which is readily available for the human genome from RefGene or Ensembl databases. To detect novel splicing sites different from the known junctions, one can first find novel expressed islands of reads with tools such as TopHat [8], and next add the predicted novel exons to the gene model, prior to using our tool. Other de novo assembly algorithms, as long as they generate de novo exon boundaries, and mapped back to genome coordinates, may also be used with our tool [51]–[54].

By sequencing and analyzing the MAQC samples UHR and HBR, and the breast cancer cell line MCF-7, we validated 25 gene fusions specific to MCF-7 and UHR. Of these, five were not in frame and had premature stop codons. These fusions might still deploy a negative constraint on the fused genes by increasing non-sense mediated decay (NMD) [55]. In addition, several gene fusions that occur at the genomic level might not have been detected by messenger RNA sequencing (mRNA) because their pre-cursor mRNAs would have been degraded by the NMD mechanism. Such fusions may be identified by DNA-level sequencing.

Of the 29 intra-chromosomal fusions called in MCF-7 in this study, 12 were not described in investigated literature (Table S4 in Text S2). Interestingly, most of the adjacent MCF-7 gene fusions did not fit the standard definition of “read-through” since they did not occur between last and first exons of the fused genes and in some cases they occurred in inverse order of expected transcription. This indicates that these fusions may had arisen due to trans-splicing or structural mutations such as deletions or inversions. These hypotheses may be tested by directly sequencing the DNA from these regions.

By surveying cancer cell lines with TaqMan assays, we observed that two of the MCF-7 fusions involving adjacent genes, ESR1-C6ORF97 and RBS6KB1-TMEM49 were expressed recurrently. Fusions of the ESR1 gene may disrupt estrogen signaling pathways and thus events involving this gene may be significant. RBS6KB1-VMP1 fusion was described as a recurrent event recently by another group [45]. VMP1 is another name for TMEM49. Amplification of the RPS6KB1 loci (Ribosomal protein S6 kinase beta-1) was described in other breast cancers as an oncogene event [56]. Still, it is possible these recurrent fusions arise only in immortalized cell lines rather than being driver mutations. In fact, the ESR1 fusion tested positive in only one of the 48 clinical breast samples, while the RPS6KB1 fusion was not expressed in any of them. Of interest, six of the fusions originated on the band 17q23, which was previously identified as a common region of amplification in cancer [57].

In many of the MCF-7 fusions, the first or early introns of the 5′ genes harbored the gene fusion breakpoint. A similar pattern was observed in prostate cancer: the complete exon-1 of TMPRSS2 was identified to fuse with ETV1 or ERG as one of the most recurrent rearrangements [31]. Recent studies on prostate cancer found extended breakpoints at the androgen receptor binding sites possibly due to LINE-1-induced ORF or topoisomerase-II beta. These enzymes, when co-recruited with an androgen receptor, were linked to increased chromosomal translocations of the TMPRSS2, ETV1, and ERG genes [58], [59]. Presence of the early 5′ breakpoints in MCF-7 genes suggest that recurrent double-stranded breaks may occur in breast tumors at the gene promoter and early splicing sites due to factors not mediated by the androgen receptor.

In conclusion, we presented a novel method of splice and fusion detection from RNA-Seq data. We sequenced MCF-7, UHR and HBR, and demonstrated high specificity in finding splices and fusions de novo. We further showed that two of the MCF-7 gene fusions are expressed recurrently in a number of tumor cell lines.

## Materials and Methods

The instruments and reagents used in this study are for research use only and not intended for diagnostic procedures. Additional methods are provided at *Supplementary Information Online*.

### RNA-Seq alignment pipeline

Reads were aligned to a reference using the Mapreads module of the BioScope 1.3 and LifeScope 2.0 software (http://www.lifetechnologies.com/lifescope). Four fasta references were used for increased throughput and accuracy: (1) genomic reference, (2) junction reference, (3) exon reference, and (4) filter reference (Figure 1B). Filter reference contained polyA, polyC, polyG, polyT, ribosomal RNAs, tRNAs, LINE, SINE, LTR and satellite repeats, rRNA, scRNA and snRNAs, as well as adaptor, barcode, and primer sequences. In our experiments, most reads filtered to ribosomal RNAs and merged adaptor-barcode-primer sequences. When aligning reads to the genome, two mismatches were allowed on the seed, and alignments were extended when possible based on a dynamic scoring function. The junction reference library was generated from a list of known and putative exon-exon pairs within RefSeq transcripts and contained approximately two million fasta entries. Reverse reads in our experiments were shorter than forward reads (25 vs 50, or 35 vs 75). To increase the mapping rate for the shorter reverse reads, they were additionally aligned to an ‘exon reference’ by allowing three mismatches on the seed. This exon reference contained each known exon as a separate reference entry. An exon rescue step was performed for reads where one pair was mapped within a gene and its pair was unaligned, by aligning the unmapped read within the downstream exons of the same gene with up to six mismatches. The genome, exon, junction, and rescued alignments were merged to generate a single set of alignments for the forward and reverse tags separately. Reads that aligned confidently to the filter reference were subtracted from these alignments. A final pairing step was performed to find most probable alignment pairs and assign a pairing quality value (for formulas see Methods in Text S1). These final paired alignments were put in a genome-coordinate BAM file which represents the summary of mapped alignments except for fusion alignments found by SASR.

### Suffix Array Spliced Read (SASR) fusion finder

For reads that were admissible as a candidate to be spliced on a fusion junction (see Methods in Text S1 for admission criteria), we performed a suffix array search as follows. A read was defined to provide evidence of a splice junction between an exon X and exon Y if and only if (1) exon X maps to the prefix of the read, (2) exon Y maps to the suffix of the read and (3) the sum of the two map lengths is equal to the length of the read. For 50-bp long reads (or 49 colors plus a leading base), the suffix data structure was simply a list (an array) of all suffixes of length 10 through 38 from all exons. The suffixes were stored in lexicographically increasing order. A string *s = s_1_s_2_…s_m_* is lexicographically (i.e. alphabetically) less than a string *t = t_1_t_2_…t_n_* if *s_1_<t_1_* or *s_1_ = t_1_*, and string *s_2_s_3_…s_m_* is lexicographically less than string *t_2_t_3_…t_n_*. Each suffix was represented compactly by a pair of integers (an integer and a byte in the implementation): an index to the relevant exon in the input exon list, and the length of the suffix. Such a data structure is called a suffix array [60]. Because of the lexicographic order proper, all suffixes that start with any given decamer were consecutive in such a list. Therefore, one may quickly find all matching suffixes with a binary search into the suffix array. Once the list of exons that mapped to the prefix and suffix of the read were identified, it could be determined whether the read provided evidence for a unique junction.

### Junction evidence graph and evaluation filters

A read was considered to be a SPAN evidence for a junction X-Y between two exons if it was already junction mapped or if it was discovered by SASR as described above. A paired-end read was considered BRIDGE evidence for a junction X-Y if one read of the pair mapped to exon X and the other mapped to exon Y with PQV>10.

Candidate junctions were stored, each with a count of evidence, number of unique start points and corresponding PQV, in a sparse, directed graph. In this graph, exons corresponded to nodes, and SPAN and BRIDGE evidences corresponded to two types of edges between nodes. After all evidence was collected, junctions were called by evaluating each candidate and assigning a junction confidence value. At least one and two unique evidences of each type were respectively required to call same-gene and different-gene junctions (fusion). Exons could partially overlap, allowing for junctions with different donor and acceptor sites to be counted as alternative splices as long as at least two alternatives were detected. Genes with overlapping annotations were not counted towards a gene fusion if the evidence was ambiguous.

### Paired-end RNA library preparation and sequencing

Human Breast Adenocarcinoma (MCF-7) Total RNA and FirstChoice® Human Brain Reference RNA (HBR) were obtained from Ambion. Universal Human Reference Total RNA (UHR) was obtained from Stratagene. Oligo(dT) selection was performed twice by using MicroPoly(A)Purist™ kit (Ambion) according to the manufacturer's recommendations. After polyA selection, 500 ng polyA RNA was fragmented using RNase III. 50 ng fragmented RNA was then subjected to hybridization and ligation using the SOLiD Total RNA-Seq Kit (Ambion) according to the manufacturer's instructions. Duplicate libraries, with three different insert sizes (100–200 bp, 100–300 bp, 150–250 bp), were generated from HBR and UHR RNAs. A total of 12 libraries were multiplexed using the SOLiD RNA Barcoding Kit (Applied Biosystems) and pooled at an equi-molar ratio. Two libraries were made from same lot of MCF-7 polyA RNA with standard insert size (100–200 bp). The final purified products were quantitated using a NanoDrop® instrument, and the size range of the products was confirmed by Bioanalyzer™ instrument analysis. The samples were then diluted and used for emulsion PCR. Libraries were sequenced utilizing 50 or 75 bp forward and 25 or 35 bp reverse paired-end sequencing chemistry on the SOLiD system [47].

### TaqMan real-time PCR assay validation

TaqMan probes and primers were designed for selected fusion targets. For each putative fusion call, the target region for assay design was composed of 200 bases around the fusion point: the first 100 from the 5′ gene exon and the second 100 from the 3′ gene exon. If either of the exons was smaller than 100 bases, the entire exon was taken but no bases from a further exon were used. Therefore, any target region had a maximum of 200 bases. These target sequences were then used to select TaqMan assay probes and primers which were ordered from Applied Biosystems. These assays were used to validate the novel fusion candidates in Universal Human Reference RNA sample (Stratagene), MCF-7 RNA (Ambion), Human Brain Reference RNA (Ambion), and a no template control sample. cDNAs were generated from 2.5 ug total RNA from each sample using the High Capacity cDNA Archive Kit and protocol (Applied Biosystems). The resulting cDNA products were diluted twenty-fold and four replicates were run for each gene for each sample in a 384-well format plate on 7900HT Fast Real-Time PCR System (Applied Biosystems).

### Cancer cell line and breast cancer clinical sample screening

24 selected fusion targets (Figure 6) were screened across 20 cancer cell line RNAs and negative template control (NTC, Table S5 in Text S2) using TaqMan probe and primers. Real-time PCR reactions were run as described above. The same selected 24 fusion targets were also screened in 48 breast cancer clinical samples (Origene) using TaqMan probe and primers. cDNAs were generated from 2 ng total RNA from each sample using the High Capacity cDNA Archive Kit and protocol (Applied Biosystems). The resulting cDNA was subjected to a 16-cycle PCR amplification followed by real-time PCR reaction using the manufacturer's TaqMan PreAmp Master Mix Kit Protocol (Applied Biosystems). Preamplifed cDNA products were diluted twentyfold and four replicates were run for each gene for each sample in a 384-well plate on a 7900HT Fast Real-Time PCR System (Applied Biosystems).

## Supporting Information

Text S1 **Supporting Methods and Figures.** This text contains additional methods and figures in support of the main manuscript.
(DOCX)

Test S2 **Supporting Tables.** This table contains supplementary tables supplied as multiple worksheets in support of the main manuscript.
(XLS)
