# Supplementary material for: RNA-Seq Mapping and Detection of Gene Fusions with a Suffix Array Algorithm
Source: PLoS Comput Biol. 2012 Apr 5;8(4):e1002464. doi: 10.1371/journal.pcbi.1002464 (PMC3320572; doi:10.1371/journal.pcbi.1002464)
Supplement: Text S1 — Supporting Methods and Figures. This text contains additional methods and figures in support of the main manuscript. (DOCX) [file pcbi.1002464.s001.docx]

**Text S1 Table of Contents**

Supplementary Methods: 2

1. Pairing Quality Value (PQV) 2

2. Annotation-Aided Alignment Rescue 5

3. Annotation-Aided Pairing 6

4. RefSeq Gene Model Filters 6

5. Suffix Array Junction Finder Admissable Reads and Data Structures 7

6. Gene Fusion Simulations 8

7. Junction Confidence Value Formulation 9

8. JCV Simulations and Predictive Power 10

Supplementary Figures: 12

Figure S1. Insert size distribution. 12

Figure S2. Gene RPKM scatter plots and correlation between libraries. 13

Figure S3. Histogram of gene coverage. 14

Figure S4. Pairing quality value increases RPKM vs. expression assay slope of correlation. 15

Figure S5. Pairwise scatterplots of log ratios for RefSeq vs PQV thresholds. 16

Figure S6. Refseq RPKM Distributions vs PQV. 17

Figure S7. Shared known and putative junctions. 18

Figure S8. JCV Simulation to test TPR and FPR rates for different misalignment ratios and expression levels. 19

Figure S9. Accuracy of JCV. 20

Figure S10. False Discovery Rate of JCV. 21

Figure S11. Simulation model for reconstructing an altered DH10B genome for testing splicing and gene fusion detection. 22

Figure S12. Known/Novel Junction Ratio for Tophat vs. Lifescope. 23

Figure S13. Comparison of unique Start Points and JCV values for MCF-7 calls. 24

Figure S14. Annotation aided read rescue. 25

Figure S15. Annotation aided PQV. 26

## Supplementary Methods:

### 1. Pairing Quality Value (PQV)

The mapping and pairing pipeline reports multiple sets of possible alignments for any given pair of reads. The pairing quality algorithm uses a Bayesian approach to calculate the quality of a given alignment for a pair of reads and the alignment with the highest pairing quality value (PQV) is chosen as the primary alignment for the pair of reads. The PQVs represent the Phred-Scaled quality score, to be useful for down stream variant detection and fusion detection. Quality of any given alignment for a pair of reads r_1_, r_2_ mapped to positions x_1_ and x_2_ in the reference genome is represented by

where A represents the event when reads r_1_ and r_2_ are sequenced from locations x_1_ and x_2_ respectively and P(A | r_1_, r_2_) is the probability of the event A occurring given the pair of reads r_1_ and r_2_. Using the Bayesian approach, the posterior probability P(A| r_1_,r_2_) is given by

The probability P(r_1_,r_2_), of finding reads r_1_ and r_2_ is a function of the complexity of the genome sequenced and for the purpose of simplicity we calculate the probability as

,

where, M is the set of all possible alignments to the reference genome for reads r_1_ and r_2_. Using this relationship to represent P(r_1_, r_2_) in the previous equation we get,

The prior probability P(A) of the event A is further given by

where, B(r_1_,x_1_) is the event that read r_1_ is sequenced from location x_1_ in the genome and P(A|B) is the conditional probability of finding the event A where read r_2_ is sequenced from location x_2_, given that read r_1_ was sequenced from location x_1_. The probability P(B) is a constant for any given read r_1_, and the conditional probability P(A|B) should theoretically follow the insert-size distribution. In cases where a pair of reads have a unique set of alignment to the reference genome, the posterior probability P(A | r_1_, r_2_) would always result in 1 thereby obscuring the relative quality of the alignment compared to those of other read pairs. To overcome this issue, we calculate a background probability P_B_ which represents the probability of finding an alignment to the reference genome with M+1 mismatches, where M is the maximum allowed mismatches set in pairing.ini file.

In case of uniquely paired reads the posterior probability is given by

For mapping using local alignment method, the likelihood function P(r_1_,r_2_ | A) is given by

where, L_1_ & L_2_ are the read lengths for reads r_1_ and r_2_ resp., k_1_ & k_2_ are the alignment lengths (k_1_  ≤ L_1_ and k_2_ ≤ L_2_), m_1_ & m_2_ are the number of mismatches and *e* is the sequencing error rate. In order to be consistent with the Phred quality score, PQV is computed as the negative log odds of misaligning the pair of reads.

The resulting pairing quality values are normalized by the maximum possible value to ensure that the pairing quality values are within the range [0,100].

PQV_max_ is the maximum possible pairing quality value when the pair of reads map uniquely to the reference with zero mismatches.

The pairing algorithm searches for gapped alignments (InDels) when one of the read in a pair, maps to the reference genome and the other read in the pair does not map to the genome within the insert-size range. If both an un-gapped and a gapped alignment are found for a given read then due the low prior probability of 10^-4 assigned to the gapped alignments the PQV for gapped alignments will be zero.

3’

5’

3’

5’

F3

R3

InDel Size

Partial Alignment

So in calculating the PQV for gapped alignments, the alternative hypothesis tested is the probability of finding the partial un-gapped alignments. The read with the gapped alignment is treated as two partial reads on either side of the InDel start point and the half with the greatest length is used as the partial alignment length for the alternate hypothesis. This ensures that gapped alignments with InDel starting point at the middle of the read with significant length of alignment on either side of the InDel starting point will be assigned a higher PQV compared to gapped alignments with InDel starting point close to either ends of the read.

For reads with multiple un-gapped alignments, the one with the highest PQV is chosen as the primary alignment for the read and is reported to the BAM file. In cases where there are multiple alignments with the same PQV, then the primary alignment is chosen in random from among the alignments with the same PQV.

### 2. Annotation-Aided Alignment Rescue

In order to “rescue” (map reads with more mismatches tolerated on specific regions) reads that map across intron junctions, an exon-based rescue scheme was implemented. For each anchor forward alignment without a reverse pair in the proper range (defined as 1 to 100,000 bases or the end of the gene), and vice versa, its pair was rescued on possible downstream exons of the same gene. Anchor alignments that mapped either on exon or intron regions of a gene were considered (**Figure S14**). A small wobble of a few bases was allowed around the exon borders when defining the rescue alignment region. For performance reasons, rescue was performed only within the rescue distance of a potential transcript (on the concatenated exons). Rescue distance was computed using the formula *mean insert size + 3 × standard deviation insert size* to account for the 99.7% of the inserts. Optionally, rescue may be performed on the entire exonic space to improve recall (sensitivity), but this may increase both the number of false positives and the run time.

### 3. Annotation-Aided Pairing

Pairing matches the forward and reverse tag alignments and determines the confidence in the paired alignment. The Pairing Quality Value (PQV) is a Phred-scaled quantitative measure of the confidence of aligning a read to the correct location in the reference genome. The PQV is represented as the negative log odds of misaligning a read *(-10log_10_[probability of error]*). The posterior probability of correctly aligning a read pair to reference was calculated using total alignment length of the paired reads, total number of mismatches to the reference, and other information such as insert size and gene annotations. Pairing quality of read pairs aligned within the same gene was not penalized regardless of their genomic distance, while alignments that fell on different genes or inter-genic regions were penalized. Paired-end alignments with the highest PQV were tagged as primary alignments ^1^. Pairs that fell within 100,000 base pairs in the same gene were flagged as proper pairs. The pairing module also considers spliced alignments when calculating the range for proper pairs as shown in **Figure S15**.

### 4. RefSeq Gene Model Filters

The human (*hg18*) RefSeq database with human transcript annotations was downloaded from the UCSC Genome Browser on May 13, 2009. The database contained 216,884 unique exons annotated on 21,808 genes. There were 2356 multi-gene exon clusters, and 293 multi-gene clusters in the annotation corresponding to exact exons shared between multiple genes, and genes sharing multiple exons, respectively. 229 of those were heterogeneous gene clusters, meaning genes that weren’t 100% identical. 41 of those contained dissimilar gene names (3chars, LOC, FAM, C#ORF# ignored). Of these, 17 clusters had genes sharing one or fewer exons, 29 clusters had genes sharing two or fewer exons and 36 clusters had genes sharing ten or fewer exons. The gene models are processed to eliminate non-unique exons and genes that overlap were clustered. This system helps eliminate many false positive junction calls that arise due to overlapping annotations.

### 5. Suffix Array Junction Finder Admissable Reads and Data Structures

SASR first iterates over existing alignments to identify reads that are admissible as evidence for spanning junctions. The criteria are as follows: (1) The read must be marked “primary”, (2) the original read length must be longer than 35 bp, (3) the read must be partially mapped (50 to 80% of the read mapped), (4) the read must not have already aligned to a spliced junction, and (5) the read must provide evidence for a junction uniquely. SASR junction finder supports color (di-base) and base reads generated by the SOLiD sequencer. In color space, two fused exons introduce an additional color between them which does not map to a reference genome. This color is not internal to either exon in the pair, so the count in condition (3) needs to have a “plus one” in color space (**Figure 2.B**).

Two data structures of 12,692,600 left- and right-end suffixes (from *hg18 RefSeq* gene models) were constructed. These suffixes were sorted lexicographically (**Figure 1.C)**. For each read that was partially mapped to the genome, the search target was a contiguous decamer “block”, defined as the sub-array of exon suffixes that begin with a 10 bp long sequence. Once a matching block was found, the rest of the match was extended within the block, with two mismatches allowed for the entire suffix alignment. A left and right alignment couple that spanned the full read was registered as evidence if there was only one such couple of exons, i.e., a non-redundant evidence (**Figure 1.D**). In order to allow mismatches in the decamer prefix, we applied a clipping scheme by iteratively removing one to ten bases (or di-bases) from the beginning and end of the read and searched suffixes that map to the clipped read. For the *hg18* exon suffixes, the blocks were reasonably small; the average block size, which was the number of times the suffix occurs in *hg18* exons, was 6.3 and more than half of its decamers, that is 538,709 out of a total of 975,292 had a block size less than six suffixes. The maximum block size was 648. This block size distribution allowed efficient screening of human exons for fusion evidence (**Figure 1.E)**.

### 6. Gene Fusion Simulations

We simulated splicing and fusion junctions on an E.coli (DH10B) resequencing dataset by introducing artificial introns (200 bases) on the genome sequence every 10,000 bases, and artificial genes at the gene model. This way we treated the original DH10B sequencing experiment as the transcriptome and the altered sequences as the genome. Each artificial continuous genomic region was defined as an exon in the GTF file supplied to the system. Every 5 exons were defined to constitute a gene. We further split the genome into “contigs”, artificial chromosomes that contain 2 genes each in order to test intra and inter-chromosomal fusions (**Figure S11**). For the entire DH10B genome, 94 genes, 470 exons and 47 contigs were defined and added to gene model, along with the new fasta reference sequence. In this simulation, there were 93 fusion junctions that are possible since reads from the original DH10B experiment would span those exon-exon boundaries in the new genome. 47 of these were intra-contig fusion junctions (meaning between two genes on the same contig), and 46 were inter-contig (meaning from the last gene in one contig to the first gene in next contig). Mapping the original reads from the sequencing experiment onto the new genome, and running fusion finder, we detected 86 out of 93 fusion junctions with both single read and paired-end evidence. BLAST analysis of the undetected fusion junctions showed that the missing regions were “multi-mapped” so were ambiguous to junction detection.

### 7. Junction Confidence Value Formulation

In order to assign a confidence level to the candidate junctions, a Junction Confidence Value (JCV) metric was defined as follows:

Equation 1.

Equation 2.

where PQV values were summed for the *i*’th unique paired read evidence of the candidate junction *j_x-y_* () where *x* and *y* are the exons of the junction. For each unique single read (junction spanning) evidence, PQV*_i_* was set to ten in order to contribute a minimal additional evidence. For multiple alignments from the same start point, PQV of one of the alignments was selected (randomly). The Error Expectation Metric (*EEM*) was used to quantify the error rate expected from highly expressed junctions. The higher the absolute proper mapped read coverage of the exons (*RC*), higher the expected error rate (product of the coverages). Our estimation further considers the length of exons, and a conservative insert range. In the equations, is the length of the exon; and are the mean and standard deviation of the insert size for the current experiment *T*. After it was calculated, a JCV larger than 100 was floored to 100, and one smaller than 0 was raised to 0. According to this metric, if a JCV approaches 100, it is more likely to be a real junction.

### 8. JCV Simulations and Predictive Power

We developed two models for true and false junctions, defined parameters for their error models and simulated 1,000,000 junctions and calculated corresponding JCVs for each permutation of the parameters. From the simulations, we calculated the true positive rate (TPR), false positive rate (FPR), accuracy and false discovery rate (FDR) for different parameter values. Our simulation model was based on two parameters: (a) junction expression ratio (JER); for real junctions between exons *x* and *y*, the expression ratio as a function of the coverage and (b) misalignment ratio (MR); for false junctions inferred between exons x and y, the false evidence ratio as a function of the coverage. As an example, JER may be 20:100 to mean for every 20 read pair covering the junction, 100 reads are expected to cover at least one of the exons. Higher JER values make real junctions more easily detected, whereas JER may be pretty low for low copy number transcripts. On the other hand, MR may be 1:100 meaning for every false junction read pair, 100 reads are expected to cover at least one of the exons. MR is expected to be lower than JER in general. Final parameter was defined as the JCV-cutoff for calling a junction. For example if JCV-cutoff was defined as 50, we called all junctions that had a JCV score equal to or above 50 and we did not call any junctions with score below 50. Exon coverage was selected as a random count between 1 and 10,000, and PQV were selected from a real distribution of 1,000,000 MCF-7 reads. Accuracy and false discovery rates of JCV from the simulation results, with changing JER and MR parameters are summarized in **Figures S8-10, and Table S6 in Text S2**

## Supplementary Figures:

### Figure S1. Insert size distribution.

150nt, 250nt and 100to300nt represent three different configurations for insert size. Mean insert size was between 100 to 120 base pairs for 150nt and 100to300nt cuts. 250nt cut resulted in larger insert sizes.


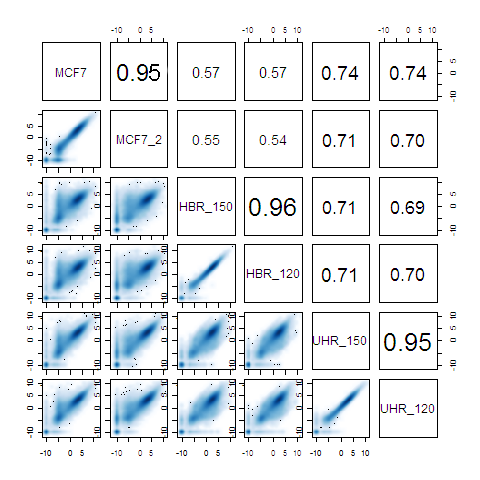


### Figure S2. Gene RPKM scatter plots and correlation between libraries.

Lower panels show the scatter plot of logarithm (base 2) of gene RPKMs between the six sequencing experiments. Data points are smoothed color density representation of the scatterplot, obtained through a kernel density estimate. Upper panels show the square of the correlation (R^2^) between two distributions.


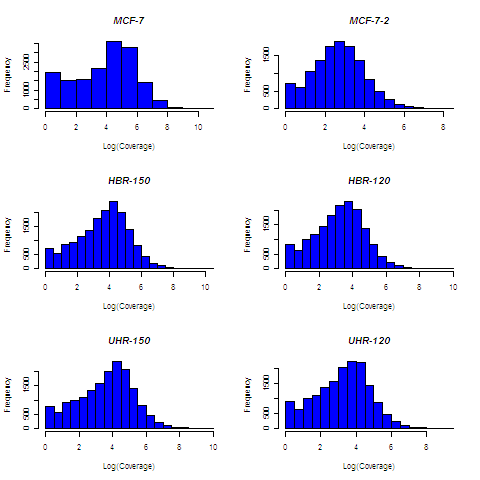


### Figure S3. Histogram of gene coverage.

Gene coverages were calculated from expressed exons of each gene with the logarithm (base 10) of total expressed reads * read length / total expressed exon length. Expressed exons were defined as exons with more than 10 mapped reads.


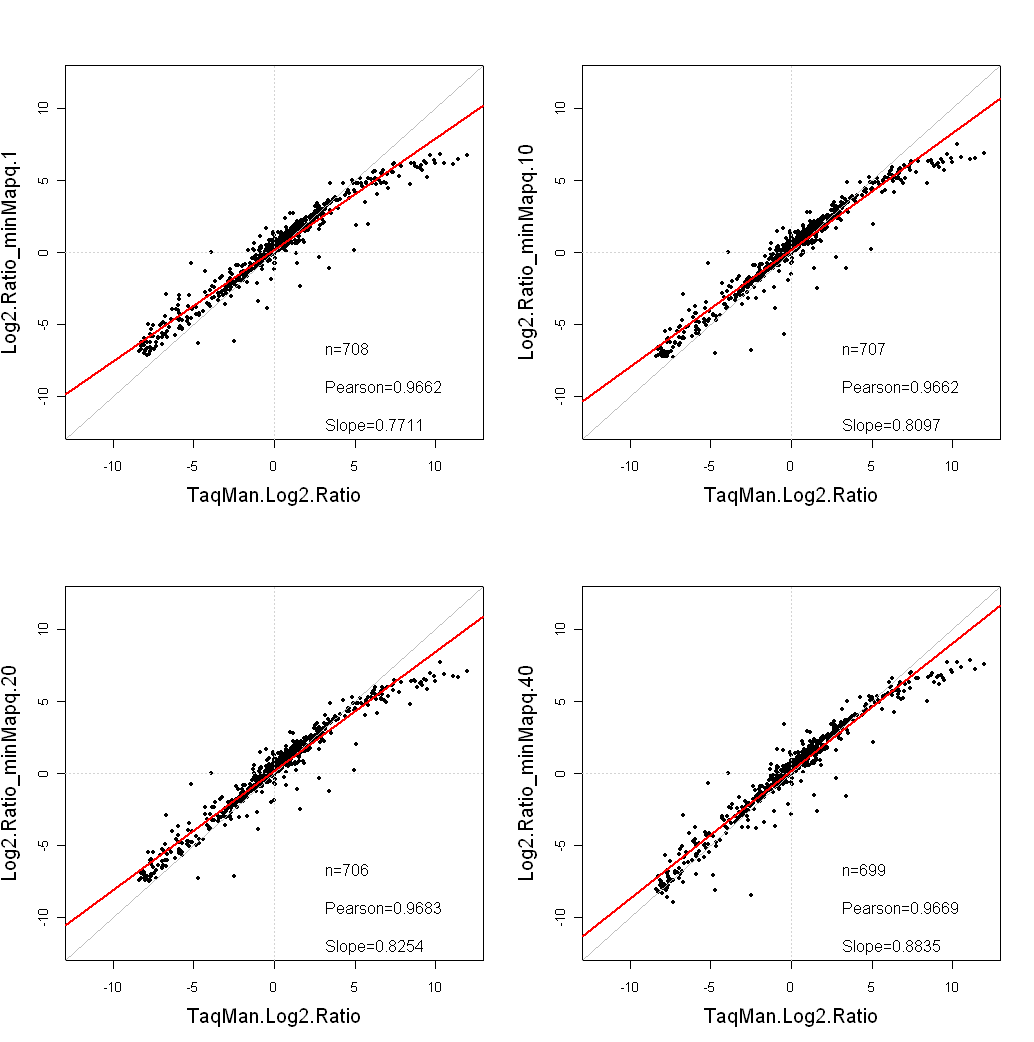


### Figure S4. Pairing quality value increases RPKM vs. expression assay slope of correlation.

The scatter plots compare the Log2(UHR/HBR) ratios of data obtained via paired-end experiments with TaqMan Real-time PCR data. Only genes called generally present in both samples for TaqMan assays were considered. minMapq corresponds to minimum PQV. Red lines indicate linear regression fits.


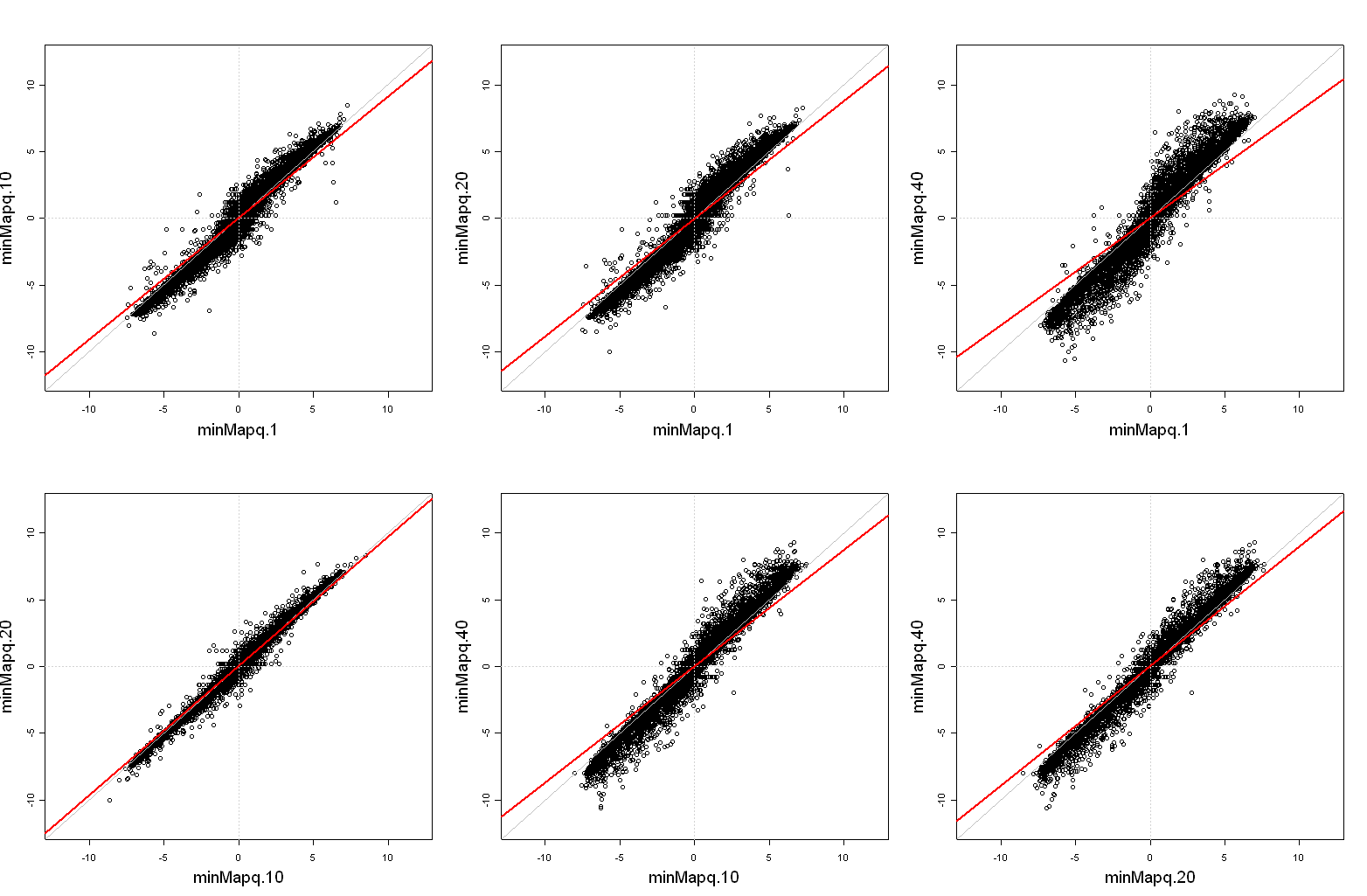


### Figure S5. Pairwise scatterplots of log ratios for RefSeq vs PQV thresholds.

Scatterplots of log2(UHRR/HBRR) expression ratios for RefSeq exons show that the slope differences seen with the TaqMan data are a global phenomena. Essentially the log ratio dynamic range increases with increasing PQV setting.


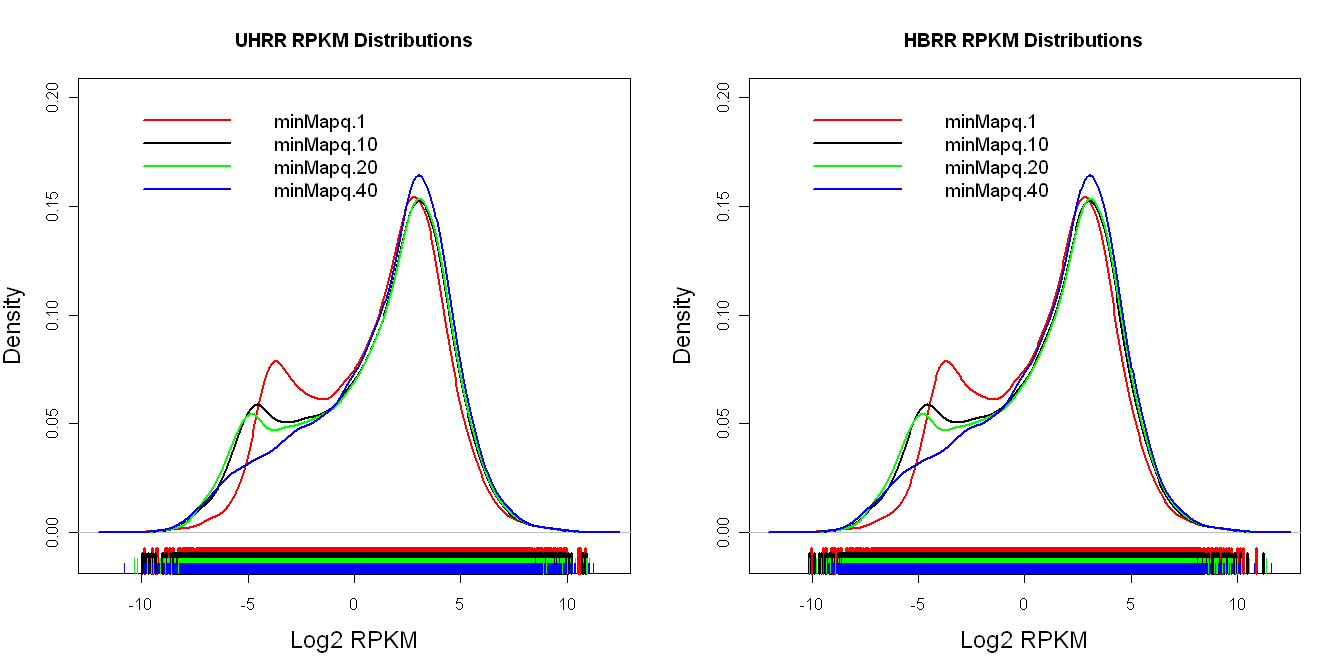


### Figure S6. Refseq RPKM Distributions vs PQV.

RPKM distributions show an increase in low end signal for lower minMapq (PQV) settings. If this increase in “sensitivity” represents additional noise this can contribute to a loss of accuracy in the fold change calculations. The increase in the low end suggests that these reads may be spurious.


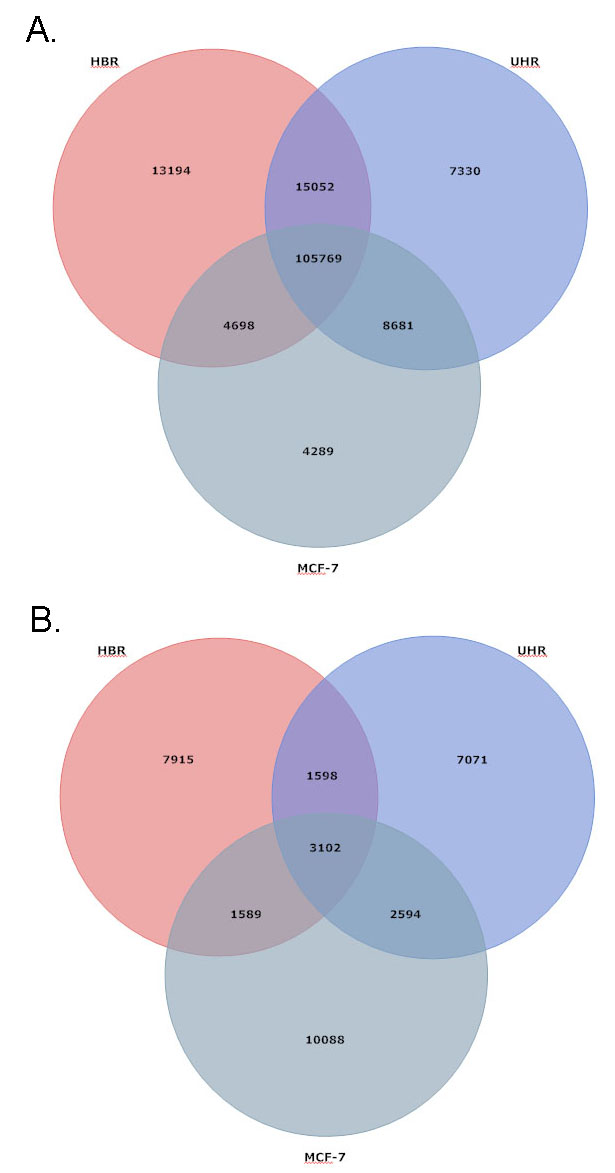


### Figure S7. Shared known and putative junctions.

**A.** Known junctions (those from RefSeq) that are shared between HBR, UHR and MCF-7. **B.** Putative junctions (those missing from RefSeq).


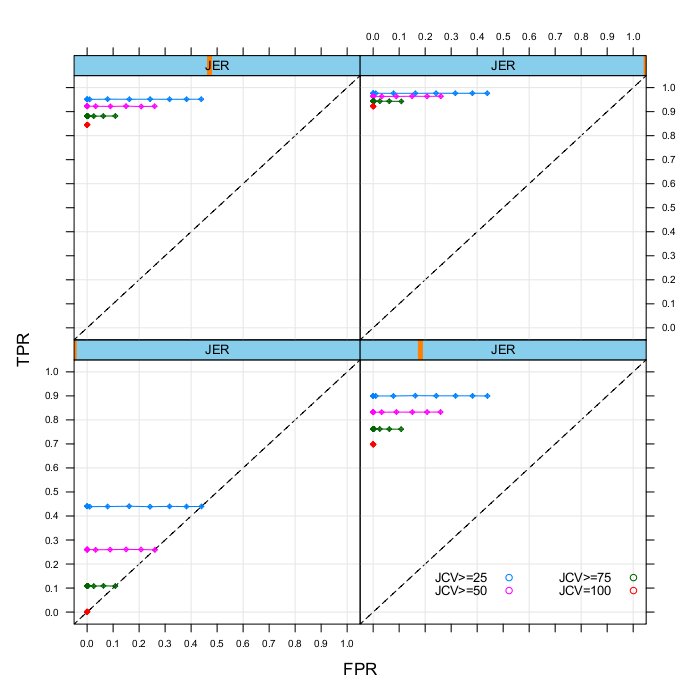


### Figure S8. JCV Simulation to test TPR and FPR rates for different misalignment ratios and expression levels.

After JCV cut-offs of 25, 50, 75 and 100 respectively are shown for blue, pink, green and red lines. Points on each line, from left to right, correspond to increasing misalignment ratios (MR) for false positive alignments (0.001 to 0.01 with 0.001 increments). Each panel shows results for different junction expression ratio (JER) values (0.01, 0.05, 0.1 and 0.2 respectively on bottom left, bottom right, top left and top right panels).

### **Figure S9. Accuracy of JCV.**

Increasing JCV values increases accuracy on the JER vs MR simulation matrix. Increasing JER and decreasing MR also increase accuracy. Color shows the accuracy value for each experiment.

### **Figure S10. False Discovery Rate of JCV.**

Increasing JCV values decrease FDR on the JER vs MR simulation matrix. Increasing JER and decreasing MR also decrease FDR. Color shows the FDR value for each experiment.


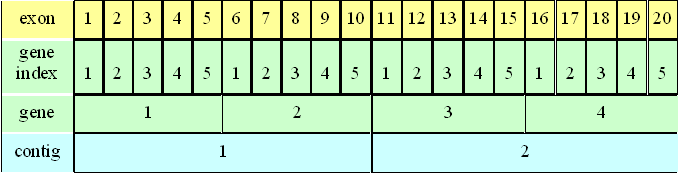


### **Figure S11. Simulation model for reconstructing an altered DH10B genome for testing splicing and gene fusion detection.**

Each continuous 10,000 base region was defined as an exon, and introns of 200 bp were introduced between the exons. Every five exon was defined as a gene. Every 2 gene (i.e. 100k bases) were defined as contigs.


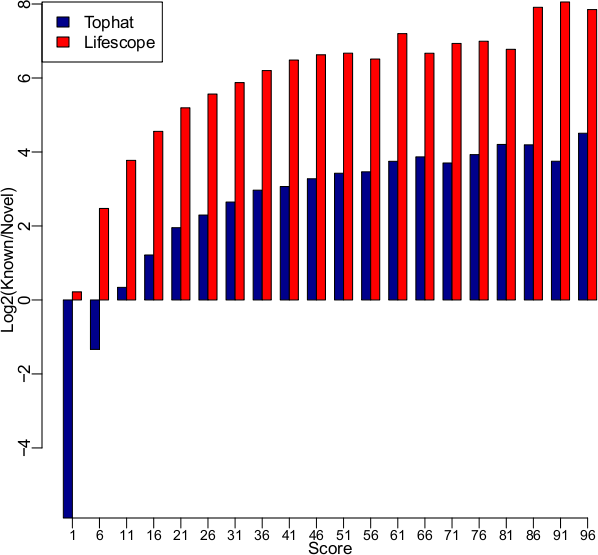


### Figure S12. Known/Novel Junction Ratio for Tophat vs. Lifescope.

Scores were determined as number of single reads spanning candidate junction for both programs. Each score bins show number of junctions with specified score to next score. Top score was 100 and any junction with score above 100 was placed to 96-100 bin. Tophat score bin raw counts: Known: 22354, 12566, 8930, 7200, 6129, 5180, 4662, 4105, 3677, 3361, 3017, 2758, 2458, 2277, 2082, 2010, 1787, 1810, 1534, 31419

Novel: 1329576, 31745, 7053, 3101, 1583, 1055, 745, 525, 439, 347, 281, 250, 183, 156, 160, 132, 97, 99, 114, 1382. Lifescope score bin raw counts: Known: 14182, 11768, 9026, 7535, 6479, 5495, 4931, 4414, 3854, 3460, 3057, 2831, 2498, 2341, 2079, 1912, 1752, 1685, 1595, 32529. Novel: 12189, 2119, 660, 320, 177, 116, 84, 60, 43, 35, 30, 31, 17, 23, 17, 15, 16, 7, 6, 141.


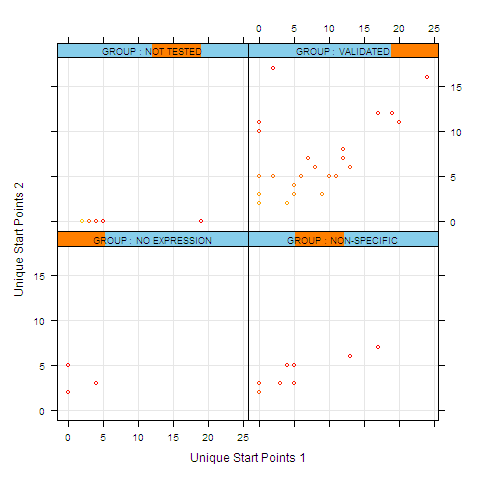


### Figure S13. Comparison of unique Start Points and JCV values for MCF-7 calls.

Comparisons are for MCF7 run 1 (x axis) versus MCF7 run 2 (y axis). Results are grouped based on Taqman validation status (i.e. Not tested, Validated, No expression and Non-specific). Color code shows the JCV (heat gradient 0 to 100 (red)).

**A:**

**B:**

**C:**

Gene A

Gene B

F3/F5

Legend:

rl

rd

rr

rd

rl

rd

rd

rr

rr

fb

rr

fb

rr

rrd

fb

rrd

rr

rl – read length

rd – rescue distance

rr – rescue region length

rrd – rescue distance on remote exon

fb – fuzzy boundary

el – exon length

rrl – rescue distance on the local exon

rr = rl + rd

rr = rrd + fb

rr = el + 2*el

rrd = rd - rrl

### **Figure S14. Annotation aided read rescue**.

**A.** For alignments that fall on an exon of a gene, and do not have a mate alignment above a certain score threshold, a special exon rescue is performed. Rescue region (rr) is defined as a rescue distance that is downstream of the alignment (the rescue distance is defined by user defined thresholds and starts from the leftmost position of the alignment for overlapping mates). The rescue region also includes certain position range in downstream exons of the same gene. This scheme helps rescue mates on different exons. **B.** If a read is mapped on the intron of a given gene, a rescue is still performed on downstream exons. **C.** If an alignment falls on intergenic region, no special exon rescue is performed. Regular rescue within pairing distance is performed.

*Spliced alignment*

**A:**

**B:**

**C:**

**D:**

**E:**

**F:**

Gene A

Gene B

F3

F5

Legend:

### **Figure S15. Annotation aided PQV.**

If a read and its pair falls on A. same exon: PQV is not penalized, B. an exon and on an intron, respectively: PQV is penalized, C & D. on separate exons of the same gene: PQV is not penalized, E. on exons from different genes: PQV is penalized. F. Spliced alignment where a read partially falls on a known gene and its pair is aligned on the exon of the same gene: PQV is not penalized.
